# Qualities of Life of Patients with Psychotic Disorders and Their Family Caregivers: Comparison between Hospitalised and Community-Based Treatment in Beijing, China

**DOI:** 10.1371/journal.pone.0166811

**Published:** 2016-11-21

**Authors:** Lizheng Guan, Yingqiang Xiang, Xin Ma, Yongzhen Weng, Wannian Liang

**Affiliations:** 1 Department of Family Medicine, School of Public Health and Family Medicine, Capital Medical University, Beijing, China; 2 Beijing An Ding Hospital, Capital Medical University, Beijing, China; 3 Department of Healthcare Reform, National Health and Family Planning Commission of P.R.C., Beijing, China; Chiba Daigaku, JAPAN

## Abstract

**Background:**

Community healthcare in mainland China is still at an early stage. The qualities of life (QOLs) of patients with psychotic disorders undergoing rehabilitation in hospitals or in the community, as well as those of their caregivers, may differ from each other.

**Objectives:**

The study was performed to evaluate the QOL of patients with psychotic disorders and assess the differences in the QOLs between patients receiving care in diverse settings (hospital vs. the community).

**Methods:**

This study was a descriptive study, in which all cases were collected from two psychiatric hospitals and five communities. Patients (n = 43) and caregivers (n = 40) in the psychiatric hospitals were grouped according to the length of illness and areas of residence and these criteria were also used to group patients (n = 55) and caregivers (n = 59) in the community. All participants were assessed using the WHOQOL-BREF (Chinese version). ANOVA was adopted to compare the QOL scores among the four groups (cases and caregivers in two settings), while confounding factors, such as age and marital status, were adjusted.

**Results:**

Among the four groups of participants, namely, hospitalised and community patients and their corresponding caregivers, community samples had a significantly lower QOL score. The QOL score for the social relationships domain of the hospitalised patients’ caregivers was significantly higher than that of the caregivers of community patients (P = 0.019).

**Conclusion:**

Community patients and their caregivers tend to have lower QOL scores than their hospitalised counterparts. The support of family members is urgently needed to provide better care for patients.

## Introduction

Psychotic disorder is characterised by distinctive distortions in thinking and perception as well as inappropriate or blunted affect. Intelligence and consciousness are usually maintained in psychotic patients, although cognitive deficits also occur [1]. No significant increase or decrease in morbidity is expected in psychotic patients. However, one study compared the morbidity of psychotic patients in Nottingham and found a slight annual increase in morbidity from 2.49 in 1978–1980 to 2.87 in 1992–1994 per 10,000 population [2], while the lifetime prevalence of psychotic disorders is estimated to be from 0.3% to 0.7% with a high recurrence rate [3].

Many patients in China had to stay in psychiatric hospitals for a very long time because of the lack of professional psychiatrists in the community. Thus, newly diagnosed patients experience difficulty in finding a hospital for treatment. Sharing tasks with community-based workers in a collaborative stepped-care framework should be considered, in particular, by integrating within national priority health programmes [4].

Numerous studies have examined the quality of life (QOL) of psychotic patients from different perspectives [5,6]. The World Health Organisation Quality of Life-BREF (WHOQOL-BREF) is one of the few instruments that can be used to evaluate subjects on four domains, namely, physical health, psychological well-being, social relationships and environment [7,8]. QOL assessment has attracted attention from clinicians, because this process evaluates the overall well-being of an individual [9]. QOL measures are especially important when treating patients with significant functional impairments [10]. QOL has been adopted increasingly in clinical trials [11]. A low QOL score can reflect the deterioration of an illness, which indicates that clinicians may need to modify treatment plans. This parameter can also be used as a factor for comparing different treatment plans and health-related costs [12,13].

Katschnig suggested that, in addition to the self-assessment of patients for their own QOL, the QOL of caregivers of these patients should also be determined for additional views on the different aspects of QOL [14]. Moreover, studies have shown that the rating of patients is poorly correlated with those by their clinicians [15–17] but are more often correlated with that from their caregivers [18]. Family caregivers are shouldering the bulk of care for psychotic patients given the context of Chinese culture and tradition, as well as considering the current health care system in the country. Therefore, patients’ QOL is highly dependent on family caregivers [19].

Studies from Western countries suggest that patients with chronic mental illness who undergo community-based rehabilitation have better outcome than those receiving long-term hospitalisation, and assessment of QOL have been used to further evaluate effect of community-based rehabilitation. Most reports on the QOL of psychotic patients in the community in China have been from Hong Kong, with few reports from mainland China. Studies in Hong Kong showed that patients who had been in long-stay care homes and half-way houses had different QOL ratings from those in hospitals [20]. We proposed that the situation would be different in mainland China, because Hong Kong is quite different from the mainland in terms of socioeconomic development and health care system [21]. Mainland China has just recently begun to offer community-based healthcare services. Thus, differentiating the QOL of patients who have been hospitalised from that of patients treated in the communities is interesting.

In mainland China, psychosis, including psychotic disorders, especially in the acute phase, is often treated in psychiatric hospitals or local mental health centres. China is currently undergoing a healthcare system reform, a major part of which is the gradual development of community healthcare services, such as establishing mental health centres in communities. However, the impact of this new development on the patients’ degree of satisfaction is still unknown [19]. The project of community mental health service supported by psychiatric hospitals has been implemented for five years. Thus, the QOL of psychotic patients, as well as their caregivers, in community should now be evaluated and compared with those of their hospital counterparts. The objectives of our study were as follows: (1) determine the differences of QOLs between hospital patients and their caregivers; (2) explore possible differences in the QOLs between community patients and their caregivers; and (3) analyse the differences in QOLs between long-term hospitalised patients and community patients.

## Methods

### Subjects

This study was a descriptive study, which included 43 hospital cases and 64 community cases. One member from each patient’s family who was the primary caregiver for more than three months was also selected for this study. Thus, 107 caregivers were also selected. Patients’ were diagnosed based on the criteria of DSM-IV and verified by two senior attending psychiatrists. All patients’ conditions were stable and cognitively sound with no language problems. Thus, the patients were able to complete the informed consent and assessments. Symptom levels were measured by the Scale for the Assessment of Positive Symptoms (SAPS) and the Scale for the Assessment of Negative Symptoms (SANS) to compare the patients’ symptoms between the two settings. The total score for the SAPS or SANS can provide overall indices of positive and negative symptoms. The study period was from March to December 2009. Informed consents were obtained from all patients and their caregivers. The research protocol was reviewed and approved by the Ethics Committee at Beijing Anding Hospital.

### Hospitalised patients and their caregivers

Hospitalised patients (n = 43) and their caregivers were selected from two psychiatric hospitals in Beijing. Inclusion criteria were as follows: met DSM-IV diagnosis of psychotic disorders; had been hospitalized for at least three months; and had been clinically stable. Ten patients were adjusted for the lengths of illness, as follows: (1) <1 year; (2) ≥1 year, but <5 years; (3) ≥5 years, but <10 years; and (4) ≥ten years. The family caregivers were also selected in the same ratio. The city to country-dwelling ratio of patients and their family members was 1:1. We received 43 copies of valid patient questionnaires, whereas 40 copies of valid questionnaires were received from the family caregivers (93.02% response rate).

### Community patients and their caregivers

Patients (n = 64) living in communities were selected from five districts, and a member from each patient’s family was also selected to match. The criteria for enlistment were as follows: met DSM-IV diagnosis of psychotic disorders and had been clinically stable. Sixteen patients were selected and categorized based on the lengths of illness, as follows: (1) <1 year; (2) ≥1 year, but <5 years; (3) ≥5 years but <10 years; and (4) ≥10 years. Their family caregivers were also selected in the same ratio. The city to country-dwelling ratio of the patients and the family caregivers was 1:1. A total of 55 (85.9% response rate) and 59 (92.2% response rate) valid questionnaires from patients and their families were received, respectively.

### Ethics Statement

This study was approved by the Medical Ethics Committee of Beijing Anding Hospital, Beijing, China. Selected patients and their caregivers were adults aged over 18 years and signed informed consent to participate in this study. Then, the participants were given an anonymous questionnaire survey. Everyone was interviewed individually, and each participant answered the questions. Thus, a surrogate consent procedure was not necessary. Anonymity and informed consent were guaranteed prior to participation in the study, and no sources of potential harm to the participants were apparent. According to the terms of agreement with the participants, personal data were not publicly available other than age, gender, marital status, education level, employment status, personal income, length of psychiatric disorder and insurance status. These data were gathered with consent forms.

### Psychiatric care

Hospitalised patients can receive regular treatment and rehabilitation. Psychiatric hospitals cannot accommodate many patients, so stabilised patients are usually discharged to their families. Psychotic patients living in communities are mostly cared for by family caregivers. Doctors in community service centres are responsible for monitoring the psychological conditions of these patients, while the local mental health centres provide clinical support. However, usually only one part-time or full-time psychiatrist is assigned at each Community Health Service Centre. This psychiatrist is responsible for all patients in the entire community with a wide variety of mental health conditions. Hence, little time is allotted for follow-up on each patient. Some staff members are rather inexperienced and not well trained, and this factor could affect the delivery of effective interventions, such as medications.

### Measuremets

#### World Health Organisation Quality of Life-Brief Form

The QOL was measured by the Chinese version of the WHOQOL-BREF, which is a 28-item questionnaire. The WHO QOL Assessment (WHOQOL) is a generic QOL instrument that was designed for people under different circumstances, conditions and cultures [22,23]. WHOQOL-BREF can be used to generate scores on four domains, namely, physical, psychological, social relationships and environmental domain. All four domains use a positive scoring system, such that the higher the score, the better the QOL will be. Fang et al. (1999) first translated WHO-BREF into Chinese and demonstrated its reliability and validity in Mainland China. In this investigation, Cronbach-α reliability coefficient of the caregivers’ questionnaire was 0.901, and that of patients’ questionnaire was 0.928, which verified the reliability of the WHOQOL-BREF.

### Data analysis

The survey data were analysed using the Statistical Package for the Social Science (SPSS, Version13.0). Social demographical factors were analysed using percentage, mean and standard deviation. Differences in gender, age, marital status, educational level, employment status, personal income, length of psychiatric, scores of PANSS and insurance status among groups were examined using ANOVA, χ^2^ analysis, t-test or Wilcoxon W. Analysis of covariance (ANCOVA) was then employed to control the confounding effects of significant social demographic factors on QOL. Bonferroni’s comparison was then used to adjust for post hoc multiple comparisons.

## Results

### Characteristics of general subjects

The study sample was composed of 43 hospitalised patients (S1), 40 hospitalised patients’ family caregivers (C1), 55 community patients (S2) and 59 community family caregivers (C2). The matching process resulted in the absence of significant difference in age, sex distribution, marital status, education level, employment status and personal income between S1 and S2. In addition, the two groups of patients with similar lengths of psychiatric illness were also selected. Thus, the mean scores of the positive and negative symptom scale (PANSS) between the two groups had no significant difference (t = 0.519, p>0.05) (Table 1).

**Table 1:** Description and comparison of social demographic variables of the sample.

	Psychiatric Hospital		Community		Test
	Patients (S1)	Family caregivers(C1)	Patients(S2)	Family caregivers(C2)	
	N = 43	N = 40	N = 55	N = 59	
Age(years)	37.2±12.7	48.2±14.4	37.8±15.5	49.3±14.1	F = 10.160*
Gender percentage (%)					X^2^ = 1.725
Male	37.2	48.7	43.6	49.2	
Female	62.8	51.3	56.4	50.8	
Marital status (%)					X^2^ = 44.852*
Not married	65.1	15.8	47.3	8.8	
Married	34.9	84.2	52.7	91.2	
Education level (%)					X^2^ = 6.398
Illiterate or Primary School	0	2.5	10.9	6.8	
High school	65.1	42.5	58.2	57.6	
University or above	34.9	55.0	30.9	35.6	
Employment status (%)					X^2^ = 7.735
Unemployed	43.9	15.4	34.0	32.7	
Employed	56.1	84.6	66.0	67.3	
Personal income (dollars per month)					Z = -0.168
median	191.9	--	159.9	--	
range	0~1279.2	--	0~1279.2	--	
Length of psychiatric illness (years)					Z = -0.004
median	4.5	--	4.5	--	
range	1~38	--	1~33	--;	
Scores of PANSS	57.1 ±18.2	--	55.2 ±16.7	--	t = 0.519;d.f = 96
Insurance status (%)					X^2^ = 2.403
Without insurance	34.9	35.0	40.0	44.1	
Medical insurance	41.9	35.0	38.2	37.3	
other funding	23.2	30.0	21.8	18.6	

Note. S1 = Hospitalised psychotic patients; C1 = Hospitalised psychotic patients’ family caregivers; S2 = Psychotic patients in the community; C2 = Psychotic patients’ family caregivers in the community; PANSS = positive and negative symptom scale

* P<0.001

We also compared the demographic variables of the four groups. The patients’ mean age is 37.5 years (SD = 14.3). Moreover, 59.2% of the patients were female, 61.2% had an educational level of middle school, 55.1% were single and 59.2% were currently employed. The family caregivers’ mean age was 48.9 years (SD = 14.1). Additionally, 50.5% of the family caregivers were female, 51.5% had an education level of middle school, 84.8% were married and 70.7% were employed. The patient’s age was usually lower than that of the care giving family member. Details on the social demographic factors are shown in Table 1.

### Effects of patient’s condition on the qualities of life of patients in different settings

Despite the absence of significant difference in the length of psychiatric illness and scores of PANSS between the S1 and S2 groups, the patient’s conditions were important factors influencing the QOL of patients according to some studies [24]. ANCOVA was used to examine the difference in QOLs between patients in different settings (Table 2).

**Table 2:** Analysis of covariance (ANCOVA) and different interaction between different groups and patient’s conditions among four domains.

	Qol-Phy	Qol-Psy	Qol-Soc	Qol-Env
	F	F	F	F
Groups^ Length of illness	2.225	3.038	0.006	4.876*
Groups^PANSS	0.383	0.469	0.346	0.270
ANCOVA	29.033*	9.796*	10.266*	0.101

Note.

^ = interaction; Phy = Physical health; Psy = Psychological health; Soc = Social relationships; Env = Environment

* P<0.05

Table 2 shows that S1 and S2 groups showed diverse scores in all QOL domains, except for the environmental domain. Detail scores in the four domains are described in the following tables.

### Effects of demographic factors on qualities of life of patients and caregivers in different settings

Multivariate ANOVA (MANOVA) was used to examine whether the dependent variables could be grouped together for analysis. Different social demographic factors were tested first with MANOVA. If the data did not meet the criteria for multivariate analysis, ANCOVA and Bonferroni were used to compare the four groups.

First, we used Box’s Test of Equality of Covariance Matrices to examine whether significant differences exist in the four domains, and the results show that the dependent variables differed significantly (F = 1.433, p = 0.016). ANCOVA was employed when the data did not meet the requirements of the assumption for MANOVA, which should have the equality of covariance matrices for the dependent variable.

Analytical results show that age and marital status significantly differed from other factors, but no significant differences were found in gender, educational level, employment status and insurance status. For ANCOVA, age was set as a covariate and marital status acted as the nominal variable (Table 3). No correlations were found between the scores in the four domains of QOL with demographic variables and residential settings.

**Table 3:** ANCOVA and different interactions between various groups and social demographic factors among the four domains.

	Qol-Phy	Qol-Psy	Qol-Soc	Qol-Env
	F	F	F	F
Groups^Age	0.775	0.723	0.666	0.945
Groups^Mar	0.330	0.736	0.871	1.185
ANCOVA	11.302*	5.644*	10.93*	3.038*

Note.

^ = interaction; Mar = Marital status; Phy = Physical health; Psy = Psychological health; Soc = Social relationships; and Env = Environment

* P<0.05

ANCOVA revealed that the score differences between the groups were statistically significant for the physical (d.f. = 3, F = 11.30, P<0.001), psychological (d.f. = 3, F = 5.644, P = 0.001), social relationships (d.f. = 3, F = 10.93, P<0.001) and environmental (d.f. = 3, F = 3.038, P = 0.03) domains.

Table 4 shows the mean scores of the different domains according to the distinct residential settings. The patients’ average score for the four domains ranged from 11.59 to 14.02, with the caregivers’ score varying from 12.42 to 15.45. Psychiatric patients had lower QOL scores than their family caregivers. Community psychotic patients had the lowest scores in all QOL domains, except for the environmental one.

**Table 4:** Comparison of QOL scores among patients and caregivers of different residential settings in four domains.

	Psychiatric Hospital		Community		Multiple comparisons
	Schizophrenicpatients (S1) N = 43	Family caregivers (C1) N = 40	Schizophrenicpatients (S2) N = 55	Family caregivers (C2) N = 59	
Qol-Phy	14.02±2.56	14.44±2.96	11.59±2.85	13.86±2.64	S1vsS2;C1vsS2;C2vsS2
Qol-Psy	13.85±3.17	14.20±2.47	12.29±2.64	14.12±2.67	S1vsS2;C1vsS2;C2vsS2
Qol-Soc	13.69±3.13	15.45±2.68	11.60±3.07	13.67±3.07	S1vsS2;C1vsS2;C1vsC2;C2vsS2
Qol-Env	13.54±2.83	13.47±2.54	12.27±2.73	12.42±2.54	P>0.05

Note. Comparisons (Bonferroni) for hospitalised psychotic patients (S1), hospitalised psychotic patients’ family caregivers (C1), community psychotic patients (S2), and community psychotic patients’ family caregivers (C2); Phy = Physical health; Psy = Psychological health; Soc = Social relationships; Env = Environment; Group significance: P<0.05

Post-hoc multiple comparisons among groups were performed, and the results are listed in Table 4. In the physical domain, hospitalised patients (P<0.001), hospitalised patients’ family caregivers (P<0.001) and community patients’ family caregivers (P<0.001) showed significantly higher scores than the community patients. Surprisingly, hospitalised patients showed no significant difference when compared with family caregivers in the hospital and in the community.

The results in the psychological domain were the same as those in the physical domain. Community patients had the lowest scores, which were significantly lower than those of hospitalised patients (P = 0.034), hospitalised patients’ family caregivers (P = 0.006) and their own family caregivers (P = 0.003).

The results in the social relationships domain also showed a similar trend. The community patients had the lowest scores compared with the hospitalised patients (P = 0.004), hospitalised patients’ family caregivers (P<0.001) and their own family caregivers (P = 0.004). Meanwhile, community family caregivers’ score was significantly lower than their hospitalised patients’ counterparts in this domain (P = 0.019).

## Discussion

We confirmed our hypothesis that the QOLs of community patients and their family caregivers would be significantly different. The survey results for community patients were consistent with earlier studies [25]. Patients showed lower scores than healthy controls, consistent with several studies that have shown a poor QOL in psychotic patients compared with healthy groups. In our study, except for the environmental domain, all other domains had significantly different scores between patients and family caregivers in different settings [26–28]. Psychotic patients had evidently worse physical functions than family caregivers, because this domain includes questions associated with pain, sleep and energy. These factors are deeply affected by the patients’ disease. Lower scores of psychotic patients in the psychological domain present more extensive problem with self-esteem and subjective well-being. Lower scores from psychotic patients in social relationships indicate more difficulties in social support, especially family support, because this domain assesses the quality of interpersonal relationships with the family, social support and sexual activity.

However, this study failed to prove the hypothesis on the significant difference between the QOLs of hospitalised patients and matching family caregivers. Several reports have also focused on the QOL of caregivers, because caregivers have to bear the main burden of the chronic illness [29]. The results showed that patients’ functional status was significantly associated with QOL [30]. Many of these caregivers were at risk of burden and psychological distress [31]. The QOL of psychotic patients or caregivers have been extensively investigated, but comparison of the QOLs of both groups is rarely performed. This study showed that the QOL of caregivers was the same as that of patients, which provided a new perspective, that is, when psychotic patients are well-cared for, the patients and the caregivers gain much comfort.

This study also confirmed our second hypothesis, that is, hospitalised and community psychotic patients have significantly different QOL scores. Patients with the same durations of illness who were either hospitalised or discharged to live in a community were selected for the survey. The results showed that QOL scores in the physical health, psychological health and interpersonal relationships showed significant differences. Community patients had the lowest scores in all four domains, whereas hospitalised patients scored higher QOL. These findings are different from those of Western countries. Simpson et al. [32,33] assessed patient from the district general hospital, hostel ward and group homes using Lehman's version of QOL Interview. They found that patients in the district general hospital showed the worst scores in terms of QOL, which was also shown by Pinkney [34] and Shepherd et al. [35]. Patients living in hospitals had higher QOL because of their better living conditions, better medical care and physical improvement. The community psychotic patients’ living conditions, social interactions and physical conditions needs much improvement. Thus, QOL of community patients was lower than that of hospitalised patients, probably because community patients could not gain enough help from any professional psychiatrist because nearly no professional psychiatrists are available in community health service centres. Thus, the former patients are only provided general health care, which is not sufficient. Meanwhile, the lack of sustained support for caregivers was also another factor for the low QOL.

The results obtained from Beijing, China, suggest that objective factors of rehabilitation tend to remarkably influence the QOL. In developing countries, rehabilitation work in the community and integrating mental health into primary health care are not highly successful, because community health services are not very well established. The cares that mentally ill patients receive in hospitals and in the community significantly differ. Chan et al. (2001) compared the QOL of hospitalised and community patients in Hong Kong [36]. They found that hospitalised patients and those receiving rehabilitation treatments in the community have the same subjective QOLs. However, the community mental health care system in Hong Kong is much more well-established and functional, compared with those in Beijing, where community mental health care system is rather underdeveloped and fragmented. The community services provided in mainland China still rely heavily on the care of family caregivers. Facilities, such as long-stay care homes, halfway houses and supported hostels, which are widely used in Hong Kong, are largely non-existent in China [37,38]. Thus, for places with psychotic patients, the community should set up housing facilities which have more supervision and nursing care support. In the meantime, normal social contacts, social integration and a sense of belonging in the community should be the ultimate goal to enhance patients’ subjective QOL.

We also hypothesized a difference only in the social relationships domain between the two groups of caregivers. Domains, such as physical health, psychological health and environment, did not show significant differences between the two groups (C1 and C2). Community patients usually live with family caregivers, so their relatives are also their caregivers. When general practitioners in the community cannot provide adequate support, the families would stand alone without any help, thus affecting their QOL.

In developing countries, the rehabilitation for community patients still depends mainly on family caregivers, because the community-based care facilities are still in its early stage, characterised by insufficient resources, such as funding, personnel and equipment, and without regular training programs for primary care personnel in mental health care. This phenomenon not only leads to the financial burden on the relatives of such patients but also deficiency in skill and knowledge, which in turn affects the patient care, thus affecting the patients and the caregiver’s QOL [39]. Therefore, the government should provide more support to family caregivers, such as training, assistance and establishment of special consulting telephone services. In developing countries, given the present lack of health human resources, utilisation of resources within the patients’ family support may be an important approach to enhance the QOL of patients with mental disorders [40]. Providers and organisations should understand the needs of families and be knowledgeable of interventions for families so that they can direct families to appropriate resources if they are unable to provide the family intervention themselves [41, 42]. In this study, we found that the QOL of patients and the caregiver in a community was worse than in the hospital. Many patients, because of the lack of professional psychiatrist in the community, would rather stay in a psychiatric hospital even after becoming medically stable, which leaves newly diagnosed patients with no alternative refuge. Thus, improving the psychiatric management in a community is highly important.

Limitation: The sample size of this study was small, and all patients were recruited from Beijing, the capital of China. Thus, the real situation may be worse than what was observed in this study. The duration of the patients in the hospital, hospital fees, relationship between patient and caregiver and many confounding factors should be adjusted. In this study, some measurement biases may exist because we did not test the patients’ internal factors, such as cognitive abilities and community/social functioning. Moreover, the relationship between internal factors and QOL score was also not discussed.

## Supporting Information

S1 Data Data used for analysis(n = 197). (SAV)
